# Nonlinear dynamic separation characteristics of friction pair and experimental analysis

**DOI:** 10.1038/s41598-024-59522-5

**Published:** 2024-04-16

**Authors:** Yongqiang Zhao, Huajun Chen, Wenlong Pan, Wenqing Cai, Yuhan Zhou, Baoyu Zhai, Xiangdong Ni

**Affiliations:** 1 College of Mechanical and Electronic Engineering, Shihezi University, Shihezi, 832000 China; 2 Key Laboratory of Northwest Agricultural and Rural Affairs, Shihezi, 832003 China

**Keywords:** Dynamics simulation, Wet clutch, Separation process, Friction torque, Gap recovery coefficient, Engineering, Mechanical engineering

## Abstract

This paper aims to reduce friction pair erosion of the clutch in the case of continuous shift; the dynamic separation process of the friction pair is investigated. The temperature of the friction pair, friction torque, and separation speed in the separation process are taken as the research objects, and the dynamics simulation model and finite element thermal coupling simulation model of the clutch separation process are established. The nonlinear dynamic separation characteristics of the friction pair are investigated by comparing and analyzing the effects of control parameters such as rotational speed difference, damping ratio, and lubricant viscosity on the friction torque, friction pair separation speed, separation gap, and contact stress during the separation process. The gap recovery coefficient is proposed as a response indicator for observing the separation process in response to the inability to observe the nonlinear dynamic motion of the friction pair during the separation process and to measure the end time of the separation. Finally, the clutch was subjected to a separation test. The results show that the proposed gap recovery coefficient accurately describes the separation process. The simulation model can simulate the clutch's separation and predict the trend of separation parameters.

## Introduction

Power shift technology is essential in improving vehicle performance and optimizing power transmission, mainly by controlling the engagement and separation of multiple wet clutches to achieve uninterrupted power shifts. However, in tests of powershift transmissions, it was found that continuous shifting tended to lead to friction pair erosion. Therefore, based on the creation of this problem, this paper will address the wet clutch separation process.

In the study of the engagement and separation characteristics of wet clutches^1–4^, assuming uniform separation of the friction pair, the mechanism of friction torque generation in the engagement process of wet clutches is investigated, as well as the effects of lubricant flow and spline friction coefficients on the friction torque and the separation gap. However, the influence of the nonlinear factors of the friction pair should also be fully considered in the modeling of this study, especially in the study of the friction torque; although most of the friction torque originates from the sliding process, a small portion of the friction torque originates from the nonlinear sliding motion of the friction pair^5–8^. Investigated the effects of structural parameters such as the engagement oil pressure, the number of friction pairs, and the thickness of the pressure plate on the clutch pressure variability and torque in the clutch engagement process, respectively, and concluded that increasing the thickness of the pressure plate and decreasing the number of friction pairs can improve the pressure distribution uniformity. Firstly, the number of friction pairs is associated with the range of torque transmission; secondly, some clutches are rotating in operation, and increasing the pressure plate thickness and decreasing the number of friction pairs will reduce the reliability of torque transmission, so it is insufficient to consider only the structural parameters in the study^9–11^. The effects of transmission damping coefficients, moments of inertia, and dynamic friction coefficients on clutch engagement shocks were investigated. However, during clutch disengagement, the separation of multiple friction pairs varies, and the neighboring friction pairs are also volatile; therefore, slip impacts between multiple friction pairs should be considered^12,13^. To investigate the vibration behaviors during clutch engagement. A mathematical SAE#2 test rig model was developed, confirming no correlation between vibration amplitude and contact stress fluctuation amplitude^14,15^. The dynamic engagement characteristics of wet clutches and the main parameters influencing the drag torque are studied^16–19^. The torque characteristics of the wet clutch engagement process are investigated by simulation and experimental testing methods. During the clutch engagement process, since the separation gap and oil film thickness are dynamically changing, this affects the accuracy of the research results; the effect of lubricant viscosity on the oil film carrying capacity and the effect of the separation gap and oil film thickness on the engagement torque should be considered^20–22^. Investigated the clutch control strategy, friction plate wear mechanism, material penetration, and lubricant viscosity. Finally^23–25^, analyzes the influence of contact stress distribution on the friction plate surface, speed difference, oil pressure, and lubricant flow on the temperature field of the clutch during the engagement process. In the study of the temperature field of the friction pair, firstly, the friction factor should be dynamically changed with the increase of temperature, showing a trend of increasing and then decreasing; secondly, the temperature change is not the same for the friction pairs in different positions in the wet clutch, so the temperature change of a single group of friction pairs does not reflect the overall temperature trend of the clutch.

Based on the above research and the phenomenon of friction pair erosion, the variables of friction torque, separation time, speed difference, and separation speed, which are related to temperature, are selected as the research objects of clutch separation characteristics in this paper. Under the consideration of the influence of nonlinear factors, a nonlinear dynamic simulation model of the friction pair is established; the model is not only able to calculate the nonlinear separation speed of the friction pair in the separation process and the complex collision situation but also can effectively analyze the friction torque and separation speed generated in the process of touching and sliding. To observe the nonlinear motion of the friction pair during the separation process and the motion state after separation, the separation gap recovery coefficient is proposed as a response index to observe the separation process. Because of the defects of this model in terms of temperature and flexible body, a finite element thermodynamic coupling model of the separation process is established again to analyze the temperature and stress changes of the friction pair in the motion process more accurately.

## Theoretical model analysis

### Principle of clutch construction

**Figure 1:**
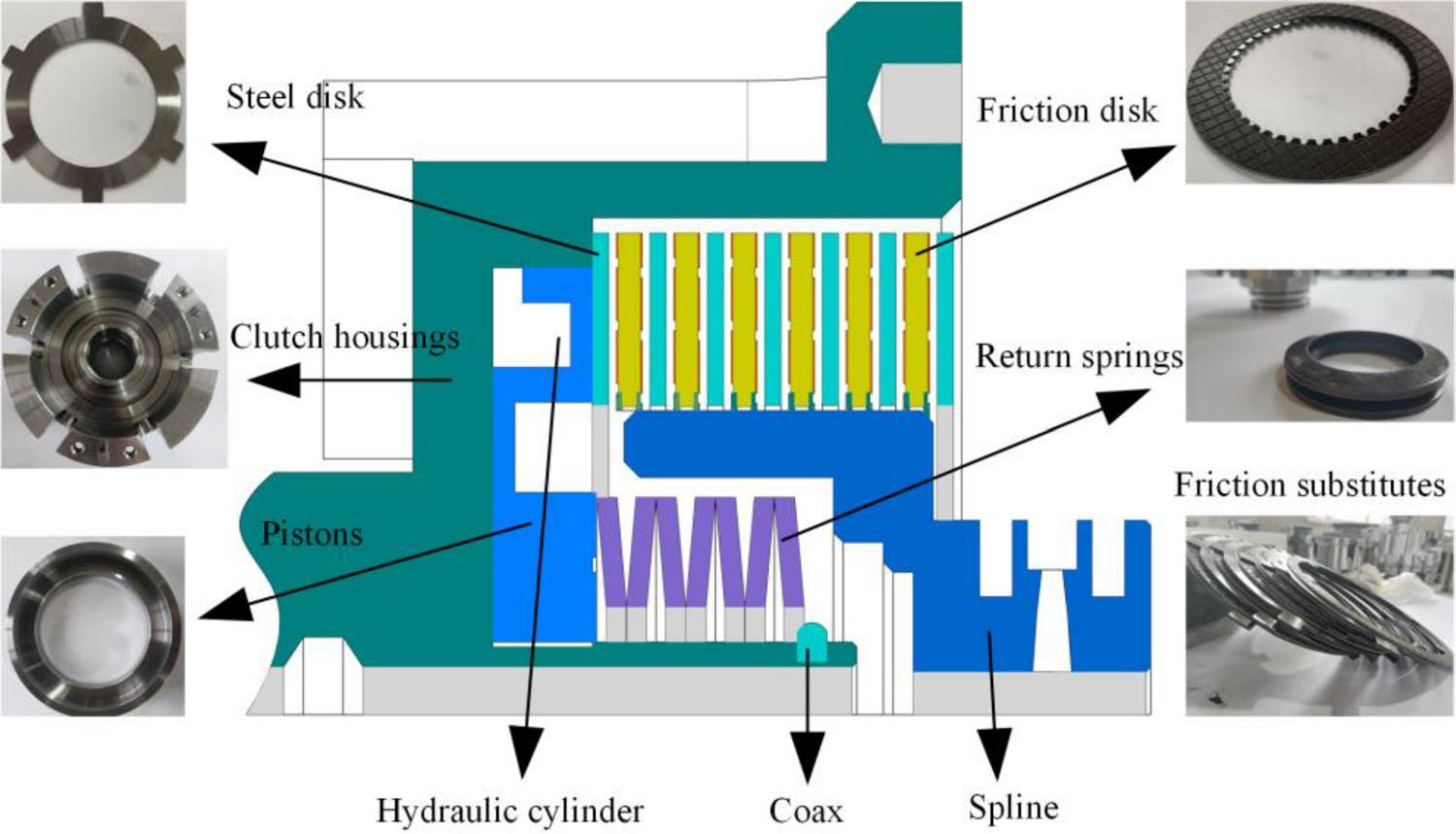
Clutch structure schematic diagram.

The structural principle of the clutch is shown in Fig. 1. During the clutch separation process, the oil pressure is firstly controlled to drop through the solenoid valve. The piston starts to reset under the push of the return spring, and the friction pair gradually starts to separate due to the decrease in piston pressure. As the pressure continues to fall, the speed difference and the separation gap gradually increase; the oil film formed in the gap pushes the friction pair to continue to separate under the action of the oil film carrying capacity until the friction pair returns to the initial position, marking the end of the clutch separation process.

### Friction pair heat generation model

**Figure 2:**
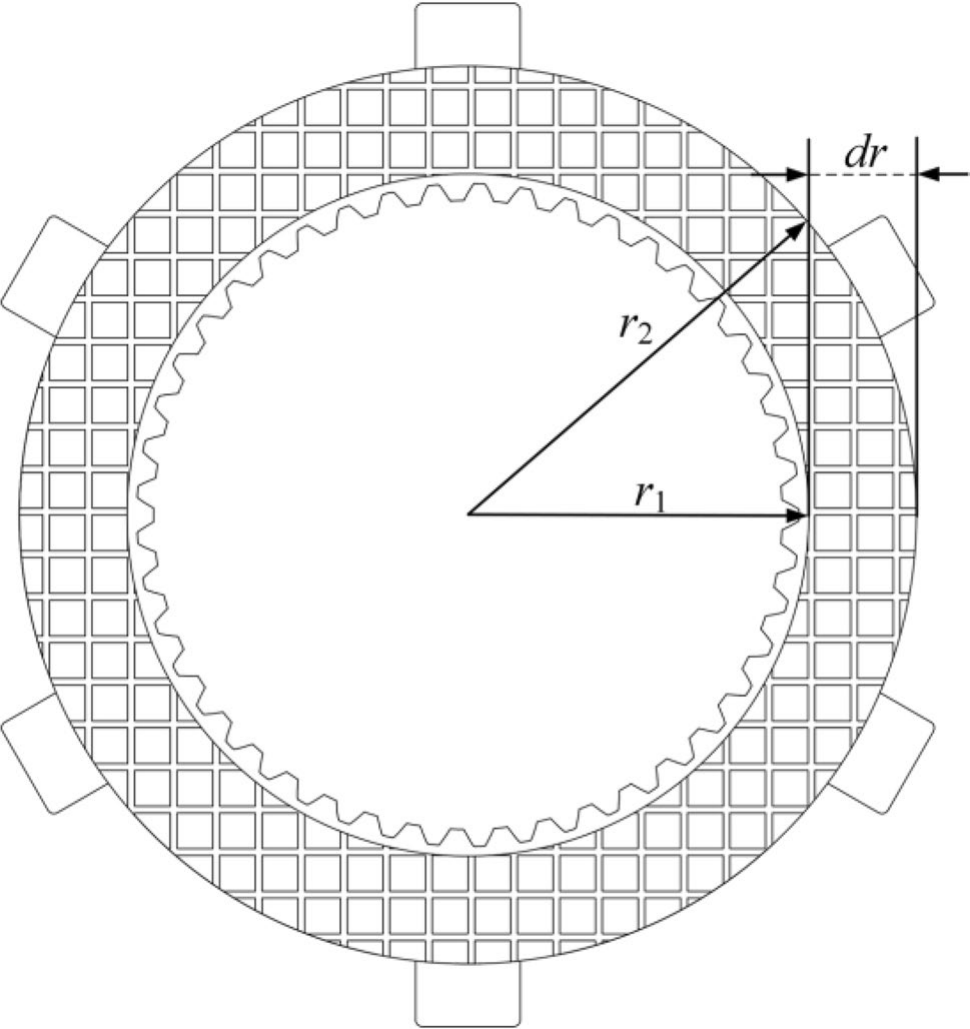
Friction diagram of friction pair.

According to the first law of thermodynamics and the principle of sliding work generation, friction versus heat is due to the rotational speed difference between the steel plate and the friction plate; mutual friction generates heat. As shown in Fig. 2, the friction diagram of the friction pair friction.

There are two mainstream frictional heat flow density calculation models based on uniform heat flow distribution and uniform pressure distribution. Among them, the pressure uniform distribution-based calculation model is suitable for thermal stress analysis. In contrast, the heat flow uniform distribution-based calculation model is more suitable for networked temperature field models.

The heat flow density model based on the uniform distribution of heat flow is calculated as^26^:

1 $$ {\text{q}}\left( t \right) = \frac{{T_{f} v_{rel} }}{{n\pi \left( {r_{2}^{2} - r_{1}^{2} } \right)}} $$

where *q*(*t*) denotes the heat flow density; *n* denotes the number of the friction pair; *T*_*f*_ is the friction torque; *v*_*rel*_ denotes the rotational speed difference of the friction pair; *r*_1_ and* r*_2_ denote the inner and outer diameters of the friction Plate, respectively.

The heat flow density model based on the uniform distribution of pressure is calculated as^27^:

2 $$ {\text{q}}\left( t \right) = \frac{{fP\left( t \right)v_{rel} r}}{{\pi \left( {r_{2}^{2} - r_{1}^{2} } \right)}} $$

where *P*(*t*) denotes the contact stress;* f* denotes the friction coefficient; *r* denotes the working radius of the friction pair. Equations 1 and 2 show that the heat flow density *q*(*t*) is related to r, n, f, and the friction torque *T*_*f*,_ the rotational speed difference *v*_*rel*_, and the time *t*. In this paper, the friction torque, speed difference, and separation speed in the separation process will be taken as the research objects of separation characteristics. The following will introduce the principle and calculation method of heat transfer of friction pair, friction torque, nonlinear time-varying slip characteristics of friction pair, and the generation principle and calculation method of the coefficient of restoration of separation gap.

### Analysis of friction versus heat conduction process

**Figure 3:**
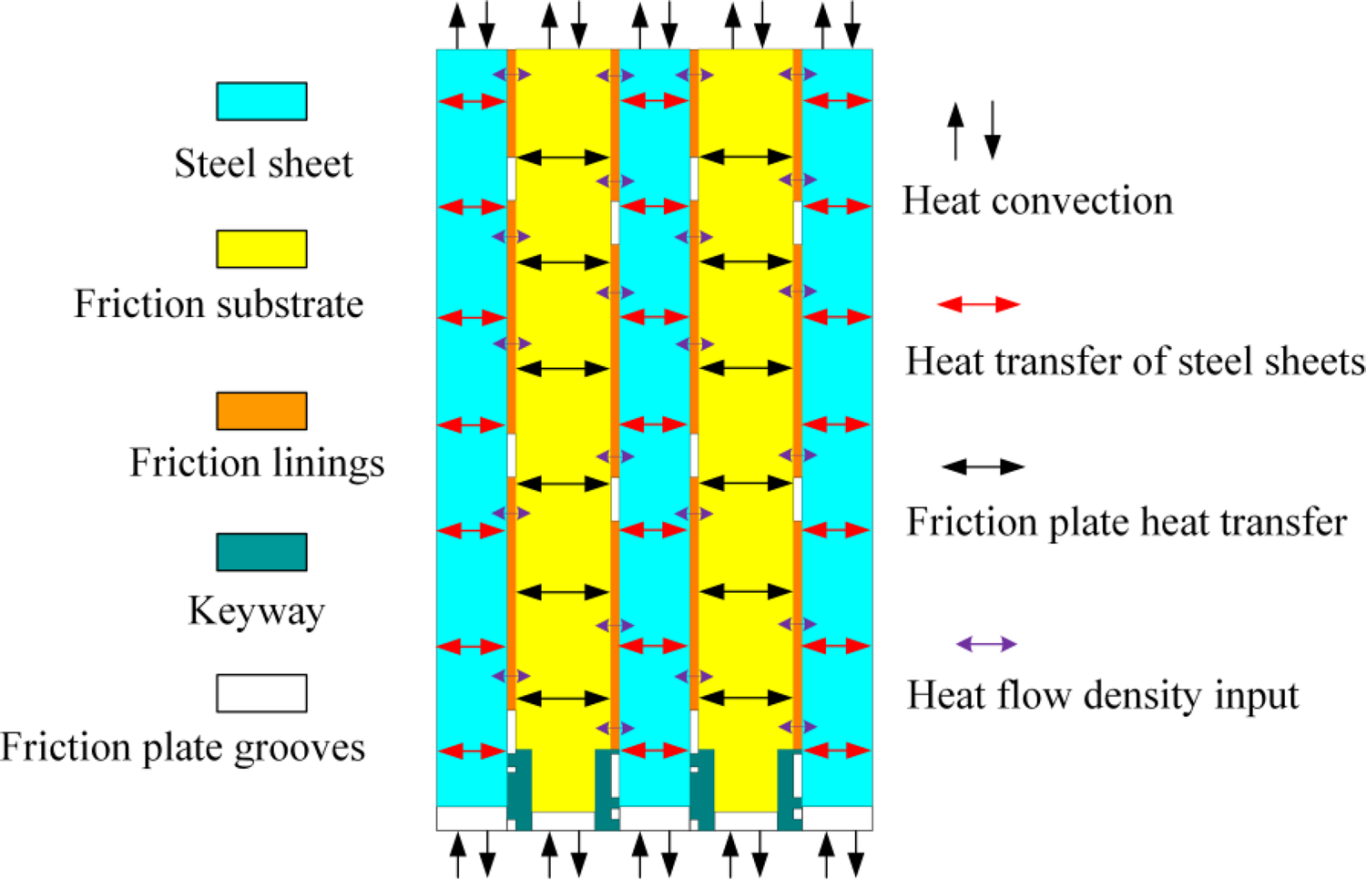
Schematic diagram of friction pair heat transfer.

The clutch is the process of engaging and disengaging a large amount of heat, part of the heat through heat conduction in the friction between the vice transfer, the other heat through the lubricating oil, clutch housing, splines, drive shafts and other components in the form of convective heat transfer to take away as shown in Fig. 3.

#### Stress concentration transfer modeling

**Figure 4:**
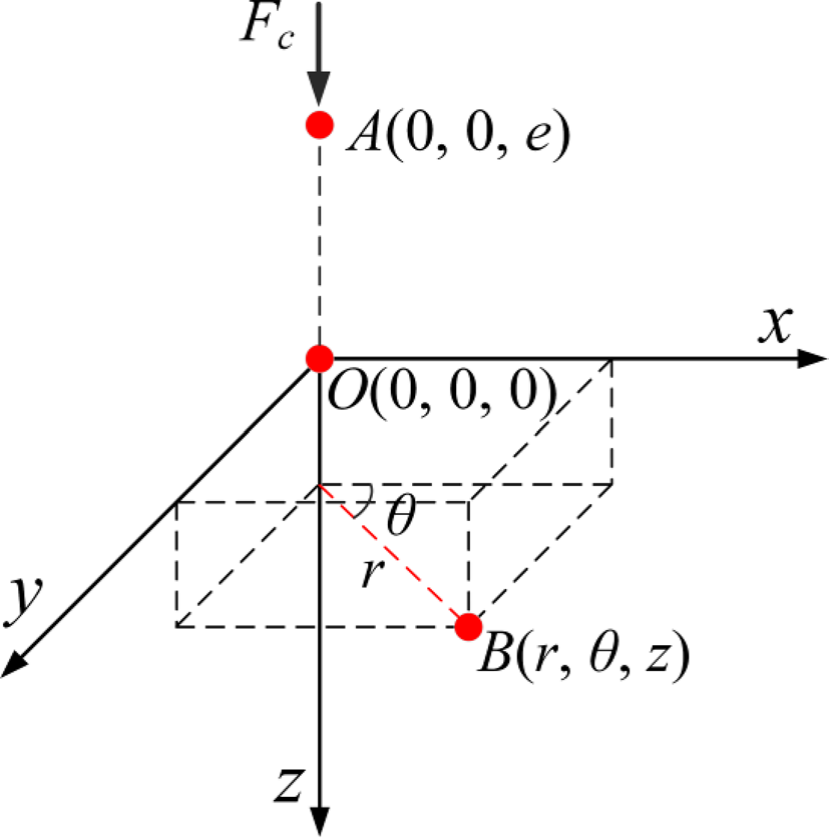
Semi-infinite contact stress transfer model.

During the operation of the clutch, there is a stress concentration between the friction plate and the steel plate. To study the transmission law of stress concentration, the semi-infinite solid column coordinate system shown in Fig. 4 is established. In Fig. 4, *Oxyz* is a three-dimensional Cartesian coordinate system, *O* (0, 0, 0) denotes the origin; *A* (0, 0, *e*) denotes any stress concentration point; *B* (*r*, *θ*, *z*) denotes any point; *r* denotes the distance from the point to the *z*-axis; *θ* and *z* are the circumferential and axial coordinates, respectively; and *F*_*c*_ denotes the concentration of the stress; *e* denotes the distance from point *A* to point *O* in z-axis direction. A concentrated stress load is applied at the coordinate point A.

From the condition of equilibrium of forces, it is obtained that:

3 $$ F_{c} = - \int_{0}^{\infty } {2\pi r\sigma_{z} } dr,z > e $$

where *σ*_*z*_ denotes the component of stress in the *z*-axis direction, through the Galyokin displacement function, the stress distribution at any point *B* in the column coordinate system can be obtained as follows^28^.

4 $$ \left\{ \begin{gathered} \sigma_{r} = \frac{\partial }{\partial z}\left[ {v\Delta Z - \frac{{\partial^{2} Z}}{{\partial r^{2} }}} \right] \hfill \\ \sigma_{\theta } = \frac{\partial }{\partial z}\left[ {v\Delta Z - \left( {1 - r} \right)\frac{{\partial^{2} Z}}{{\partial r^{2} }}} \right] \hfill \\ \sigma_{z} = \frac{\partial }{\partial z}\left[ {\left( {2 - v} \right)\Delta Z - \frac{{\partial^{2} Z}}{{\partial r^{2} }}} \right] \hfill \\ \end{gathered} \right. $$

where *v* denotes Poisson's ratio; *Z* denotes Gale kin’s function; Δ denotes the Laplace operator. When the concentrated stress acts on the origin *O*, the stress distribution in each direction can be obtained as follows.

5 $$ \left\{ \begin{gathered} \sigma_{r} = \frac{{F_{c} \left( {1 - v} \right)}}{{2\pi \left( {1 - v} \right)}}\left[ {\frac{1 - 2v}{{r^{2} + z^{2} + z\sqrt {r^{2} + z^{2} } }} - \frac{{3r^{2} z}}{{\left( {r^{2} + z^{2} } \right)^{\frac{5}{2}} }}} \right] \hfill \\ \sigma_{\theta } = \frac{{F_{c} \left( {1 - 2v} \right)}}{{8\pi \left( {1 - v} \right)}}\left[ {\frac{{F_{c} \left( {2v - 1} \right)}}{{\left( {r^{2} + z^{2} } \right)^{\frac{3}{2}} }} + \frac{{4\left( {1 - v} \right)}}{{r^{2} + z^{2} + z\sqrt {r^{2} + z^{2} } }}} \right] \hfill \\ \sigma_{z} = - \frac{{3F_{c} z^{3} }}{{2\pi \left( {r^{2} + z^{2} } \right)^{\frac{5}{2}} }} \hfill \\ \end{gathered} \right. $$

#### Heat transfer modeling

From Eq. (5), when the friction pair is equivalent to a stressed whole, the change of circumferential position does not affect the stress magnitude when the radius and axial position are constant. Therefore, the two-dimensional heat transfer equation for the friction element is established without considering the effect of circumferential position on the temperature field.

6 $$ P_{c} \frac{\partial \psi }{{\partial t}} = \kappa \left( {\frac{{\partial^{2} \psi }}{{\partial r^{2} }} + \frac{1}{r} \cdot \frac{\partial \psi }{{\partial r}} + \frac{{\partial^{2} \psi }}{{\partial z^{2} }}} \right) $$

where *ѱ* denotes the temperature; *ρ*, *c,* and *ĸ* denote the density, specific heat capacity, and heat transfer coefficient in the friction material, respectively.

The heat flux between the steel plate and the friction plate can be expressed as.

7 $$ q = \mu \left( {\sigma ,\Delta V,\psi } \right) \cdot \sigma_{a} \left( {r,\theta ,z} \right) \cdot \Delta V \cdot r $$

where *μ* (*σ*, *ΔV*, *ψ*) denotes the friction coefficient; *σ* denotes the contact stress concentration point; *ΔV* denotes the rotational speed difference of the friction pair; *σ*_*a*_ (*r*, *θ*, *z*) indicates the normal contact stress, i.e., normal contact stress.

8 $$ \gamma = \frac{{\sqrt {\kappa_{G} \rho_{G} c_{G} } }}{{\sqrt {\kappa_{m} \rho_{m} c_{m} } + \sqrt {\kappa_{G} \rho_{G} c_{G} } }} $$

where subscript *G* denotes steel plate; *m* denotes friction sheet; *q* denotes heat flux; *γ* denotes heat flow distribution coefficient.

The heat fluxes transferred to the steel and friction plates are, respectively:

9 $$ \left\{ \begin{gathered} q_{G} = \gamma \cdot q \hfill \\ q_{m} = \left( {1 - \gamma } \right) \cdot q \hfill \\ \end{gathered} \right. $$

The corresponding thermal boundary conditions for the friction pair are as follows.

10 $$ \begin{aligned} & \lambda \frac{{\partial \psi \left( {r,z,t} \right)}}{\partial r}\left| {_{{r = r_{i} }} = + h_{i} } \right.\left[ {\psi \left( {r,z,t} \right) - \psi_{e} } \right] \\ & \lambda \frac{{\partial \psi \left( {r,z,t} \right)}}{\partial r}\left| {_{{r = r_{o} }} = - h_{o} } \right.\left[ {\psi \left( {r,z,t} \right) - \psi_{e} } \right] \\ & \lambda \frac{{\partial \psi \left( {r,z,t} \right)}}{\partial r}\left| {_{z = 0} = q_{a} = \gamma \mu \sigma_{a} \left( {r,\theta ,z} \right)} \right.\omega r \\ & \lambda \frac{{\partial \psi \left( {r,z,t} \right)}}{\partial r}\left| {_{z = H} = q_{b} = \gamma \mu \sigma_{b} \left( {r,\theta ,z} \right)} \right.\omega r \\ & \psi \left( {r,z,t} \right)\left| {_{t = 0} } \right. = \psi_{0} \\ \end{aligned} $$

where *r*_*i*_, *r*_*o*_ and *h*_*i*_, *h*_*o*_ denote the inner and outer diameters and the corresponding convective heat transfer coefficients, respectively; *ω* denotes the relative angular speed of the friction pair; *t* denotes the contact time; *ψ*_*e*_ denotes the ambient temperature; *H* denotes the thickness of the friction element; *q*_*a*_ and *q*_*b*_, *σ*_*a*_ and *σ*_*b*_ denote the heat fluxes on the front and back surfaces of the steel plate and the contact stress, respectively; and *ψ*_*0*_ denotes the temperature of the initial state.

### Calculation of friction torque

**Figure 5:**
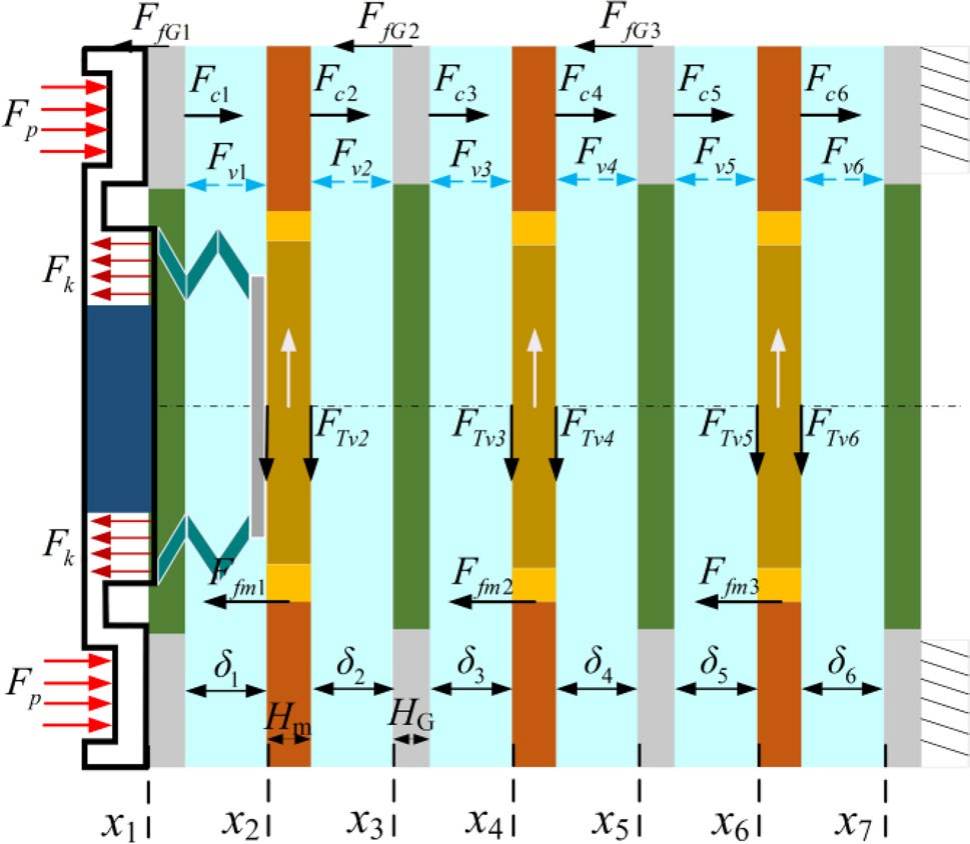
Schematic diagram of the forces on the friction pair in operation.

Clutch friction torque manifests in both engagement and separation processes, mainly relying on changing the contact stress between the friction pair to transfer torque. The friction torque in the clutch engagement and separation processes is similar in the generation mechanism, and the friction torque model derived by Greenwood and Tripp will be cited below^29^. The force on the friction pair in operation is schematically shown in Fig. 5.

The true contact area of the friction pair is *A*_*c*_:

11 $$ A_{c} = \left( {\pi \lambda R\sigma^{*} } \right)^{2} AF_{2} \left( {\frac{{\overline{h}_{t} }}{{\sigma^{*} }}} \right) $$

The effective pressure *P*_*c*_ can be expressed as:

12 $$ \begin{aligned} & P_{c} \left( {\frac{{\overline{h}_{t} }}{{\sigma^{*} }}} \right) = K^{\prime } E^{\prime}F_{n} \left( {\frac{{\overline{h}_{t} }}{{\sigma^{*} }}} \right) \\ & F_{n} \left( {\frac{{\overline{h}_{t} }}{{\sigma^{*} }}} \right) = \int\limits_{{\frac{{\overline{h}_{t} }}{{\sigma^{*} }}}}^{ + \infty } {\left( {z - \frac{{\overline{h}_{t} }}{{\sigma^{*} }}} \right)}^{n} \varphi^{*} \left( z \right)dz \\ & E^{\prime} = \frac{{2E_{1} E_{2} }}{{E_{2} \left( {1 - v_{1}^{2} } \right) + E_{1} \left( {1 - v_{2}^{2} } \right)}} \\ & K^{\prime} = \frac{8\sqrt 2 }{{15}}\pi \left( {\pi \lambda R\sigma^{*} } \right)^{2} \sqrt {\frac{{\sigma^{*} }}{R}} \\ & \varphi^{ * } \left( z \right) = \frac{1}{{\sqrt {2\pi \left( {\sigma^{ * } } \right)^{2} } }}\exp \left( { - \frac{{z^{2} }}{{2\left( {\sigma^{ * } } \right)^{2} }}} \right) \\ \end{aligned} $$

where *λ* is the roughness peak density of the micro convex body; *R* is the radius of curvature of the micro convex body; *v*_1_,* v*_2_ is the Poisson's ratio of the friction pair materials; *A* is the actual contact area, φ*(*z*) is the roughness peak probability density; *E'* is the equivalent elasticity modulus; σ* is the roughness peak variance; and *E*_1_, *E*_2_ is the elasticity modulus of the friction pair material.

*P*_*c*_ can be expressed as:

13 $$ \left\{ \begin{gathered} P_{c} \left( H \right) = K^{\prime}E^{\prime} \times 4.4086 \times 10^{ - 5} \times \left( {4 - H} \right)^{6.804} ,\quad H < 4 \hfill \\ P_{c} \left( H \right) = 0,\quad {\text{H}} \ge 4 \hfill \\ \end{gathered} \right. $$

Combined with Eq. (5), this leads to

14 $$ \left\{ \begin{gathered} {\text{erf}}\left( x \right) = \frac{2}{\sqrt \pi }\int_{0}^{x} {\exp - \eta^{2} d\eta } \hfill \\ \frac{{A_{c} }}{A} = \frac{{\left( {\pi \lambda R\sigma^{ * } } \right)^{2} }}{2}\left\{ {\left( {1 + \frac{{h^{2} }}{{\sigma^{2} }}} \right)\left[ {1 - erf\left( {\frac{h}{\sqrt 2 \sigma }} \right)} \right]} \right.\left. { - \sqrt {\frac{{2h^{2} }}{{\pi \sigma^{2} }}} \exp \left( { - \frac{{h^{2} }}{{2\sigma^{2} }}} \right)} \right\} \hfill \\ \end{gathered} \right. $$

By appealing to the contact model, the load-carrying capacity *F*_*c*_ is obtained:

15 $$ F_{c} = \iint\limits_{{A_{c} }} {P_{c} } $$

The friction torque can be expressed as *T*_*c*_:

16 $$ T_{c} = f_{c} \frac{{A_{c} }}{A}\int_{0}^{2\pi } {\int_{a}^{b} {{\text{P}}_{{\text{c}}} } } drd\theta $$

### Nonlinear time-varying slip model for friction pair separation

In a friction clutch, as the contact stress decreases, the spring force of the spring and the friction material first pushes the friction pair to separate for the first time; when the lubricating oil enters the separation gap, the lubricating oil-bearing force pushes the friction pair to separate for the second time. The friction pair must overcome the friction force and lubricant resistance in the separation process. Each friction pair separation speed and direction of motion are time-varying, so the phenomenon of nonlinear slip occurs.

**Figure 6:**
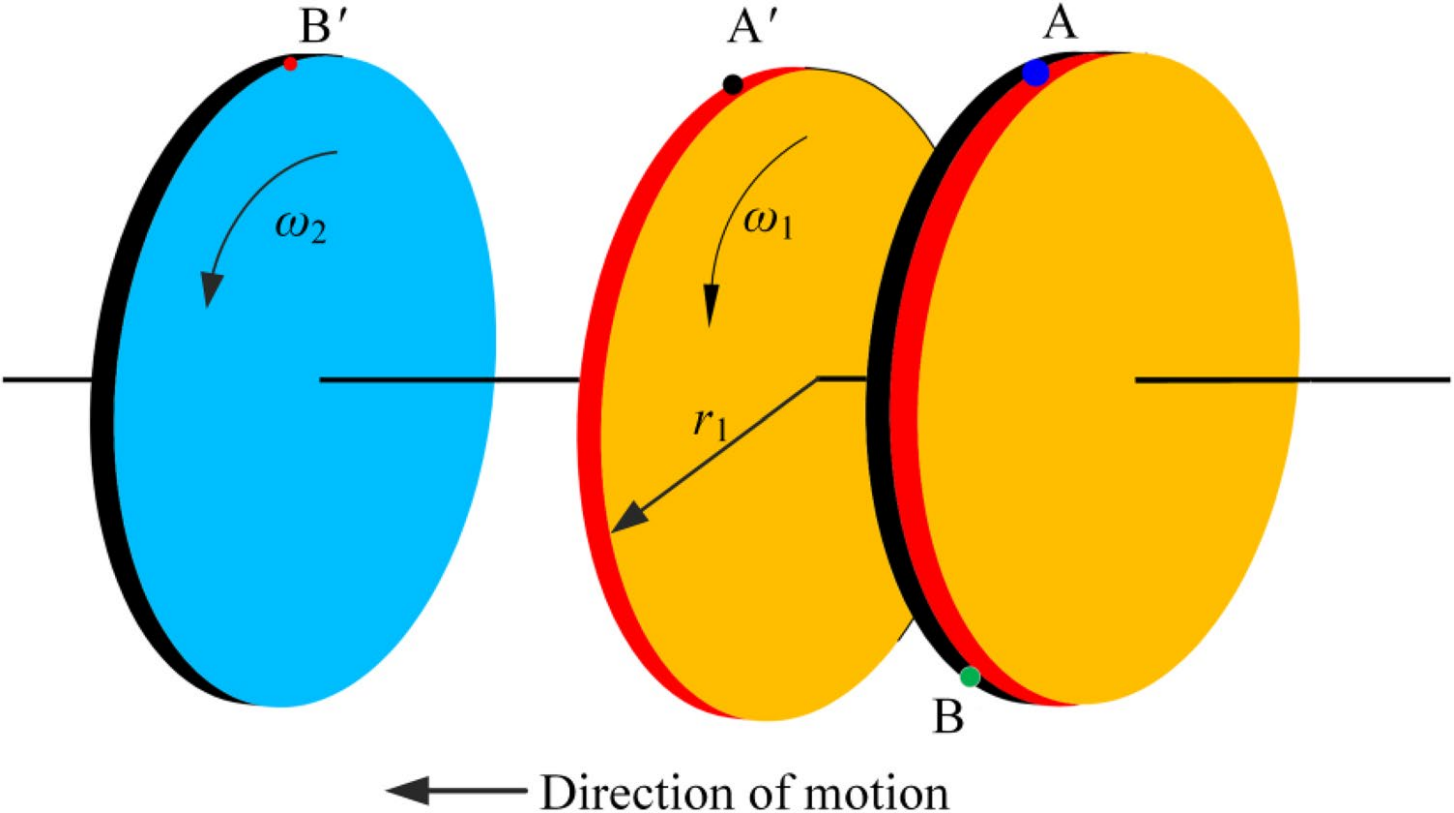
Friction pair slip model.

In Fig. 6, points A and B indicate the initial corresponding position points of the master and slave disks, respectively, and A' and B' indicate the corresponding position points after the separation of the master and slave disks has ended.

The sliding distance is approximated by the equation* L*(*t*)

17 $$ L\left( t \right) = vt = \frac{{2\pi \left( {n_{1} - n_{2} } \right)r_{1} t}}{60} = \frac{{\pi \left( {n_{1} - n_{2} } \right)r_{1} t}}{30} $$

where *L*(*t*) denotes the sliding distance of the friction plate; *n*_1_ denotes the rotational speed of the active disk; *n*_2_ denotes the rotational speed of the driven disk; *r*_1_ denotes the diameter of the friction plate; *t* denotes the separation time; and *ω* denotes the rotational angular speed.

In the separation process, the sliding distance is the distance of the movement of the friction plate and the steel plate from the engagement position to the movement when they are not engaged in the initial position. However, the friction pair separation speed and direction have uncertainty in the slipping process, and the sliding distance L(t) is obtained by fitting the speed and time equations, which are:

18 $$ L\left( t \right) = \int_{0}^{T} {\left( {\omega_{1} \left( t \right) - \omega_{2} \left( t \right)} \right)} dt\int_{{r_{2} }}^{{r_{1} }} {dr} $$

### Damping coefficient of friction pair

The attenuation of the driving force of the friction pair during its motion is called damping. When the friction pair makes a slip motion, the force that prevents the continued motion is the damping force. Moreover, the damping coefficient is the ratio of the damping force to the driving force of the friction pair motion. The damping coefficient can be expressed as:

19 $$ C = \frac{{F_{\zeta } }}{F} $$

The damping ratio can be expressed as:

20 $$ \xi = \frac{C}{{C_{0} }} $$

where *C* denotes the damping coefficient; *F*_*ζ*_ denotes the damping force; *F* denotes the driving force of the friction pair; *ζ* denotes the damping ratio; *C*_0_ denotes the critical damping. To study the effect of damping on the separation motion of the friction pair, the damping ratio is introduced as a control variable of the separation process.

### Coefficient of recovery of the clutch separation gap

At the beginning of separation, the separation gap is small, the oil film carrying capacity is low, and the temperature growth rate of the friction pair is high. When the separation gap is large, the fluctuation phenomenon of the friction pair is serious, which increases the separation shock and prolongs the separation time. Therefore, the gap recovery coefficient is proposed to characterize the fluctuation of the friction pair during the separation process and the motion state of the friction pair after separation. The separation gap is shown in Fig. 5.

The equation for the separation gap of the friction pair:

21 $$ \left\{ \begin{gathered} \delta_{1} = x_{2} - x_{1} - H_{G1} \hfill \\ \delta_{2} = x_{3} - x_{2} - H_{{{\text{m}}1}} \hfill \\ \delta_{3} = x_{4} - x_{3} - H_{G2} \hfill \\ \delta_{4} = x_{5} - x_{4} - H_{m2} \hfill \\ \vdots \hfill \\ \delta_{i} = x_{i + 1} - x_{i} - H_{jk} \hfill \\ \end{gathered} \right. $$

22 $$ j = \left\{ \begin{gathered} m,i = 2n \hfill \\ G,i = 2n + 1 \hfill \\ \end{gathered} \right. $$

23 $$ k = ceil\left( {\left\lfloor {\left( \frac{i}{2} \right)} \right\rfloor } \right) $$

where *i* denotes the separation gap sequence number; *i* $\in$[1, 2, …, 6]; *j* denotes the friction pair component; *k* denotes the upward rounding function; and *n* denotes a natural number.

The coefficient of restoration of the friction pair gap can be expressed as:

24 $$ h_{0i} = \frac{{\delta_{i} }}{{\delta_{0} }} = \frac{{x_{{\left( {i + 1} \right)}} - x_{i} - H_{{jceil\left( {\left\lfloor {\left( \frac{i}{2} \right)} \right\rfloor } \right)}} }}{{\delta_{0} }} $$

where *F*_*v*_ indicates the oil film carrying capacity; *F*_*c*_ indicates the contact force of the friction pair; *F*_*p*_ indicates the piston pressure; *F*_*k*_ indicates the spring force of the return spring; *F*_*f*_ indicates the friction force of the friction pair; *δ* indicates the gap between the steel plate and the friction plate; *G* indicates the steel plate; *m* indicates the friction plate; *a* indicates the acceleration of the motion; *δ*_0_ is the standard gap; *H*_*G*_ indicates the thickness of the steel plate; *H*_*m*_ indicates the thickness of the friction plate. The gap recovery coefficient 0 < *h*_0_ < 1 indicates that the gap is not fully recovered; when *h*_0_ > 1, it indicates transition recovery.

As seen from "Friction pair heat generation model" section, the heat generation of the friction pair is not only affected by several structural parameters. However, it is also associated with the speed difference, friction time, and torque. Therefore, this chapter chooses the rotational speed difference, friction torque, and separation speed as the research objects of separation characteristics and focuses on analyzing the friction pair heat conduction process, the friction torque calculation method, and the friction pair slip motion. Unlike the joining process, neither the friction torque nor the separation speed in the separation process can accurately measure the separation end time and the post-separation process. To solve this problem, the concept of gap recovery coefficient is proposed. Next, the friction torque and separation speed in the separation process will be analyzed by in-depth simulation based on the theory in Section II.

## Dynamics analysis of the separation process

ADAMS carried out the dynamic simulation of the clutch separation process, and four groups of friction pairs were selected as the experimental objects, with the separation gap set at 0.50 mm. The material parameters, contact conditions, and specifications of the friction pair parts are shown in Table 1, in which the values of the critical damping are obtained from the continuous testing of the model. The rest of the simulation parameters are from the powershift transmission's working parameters and related papers.

**Table 1:** Friction element material parameters.

Materials/components	Steel plate	Friction plate	Exposure	Parameters
Mass	0.680 kg	0.292 kg	Stiffness factor	1.0E+05
Inside and outside diameter	98.4 mm/132.8 mm	101.0 mm/130.9 mm	Exponent	2.2
Densities	7.80E−06 kg/mm^3^	2.26E−06 kg/mm^3^	Damping	20
Young's modulus	2.07E+05 N/mm^2^	1.0E+05 N/mm^2^	Mus static friction coeff	0.3
Poisson's ratio	0.29	0.21	Mud dynamic friction coeff	0.20–0.25
Moment of inertia lxx	9889.211 mm^4^	3976.91 mm^4^	Coefficient of restitution	0.15
Moment of inertia lzz	4934.92 mm^4^	1984.76 mm^4^	Penetration	0.1

The simulation setup time is Time = 2.0 s; the simulation step size is 8000 steps; Parasolid contact solver and GSTIFF integral solver are used. In the simulation, the value of speed difference ΔV is 180–720 rpm; lubricant viscosity η is 0.05–0.09 Pa s; and damping ratio ζ is 0.729–2.297. Comparative analysis is carried out to study the clutch separation characteristics by examining the changes in the response of three variables, namely, speed difference, lubricant viscosity, and damping ratio, to the gap restoration coefficient, friction torque, and separation speed in the clutch separation process^30^.

### Friction torque response

**Figure 7:**
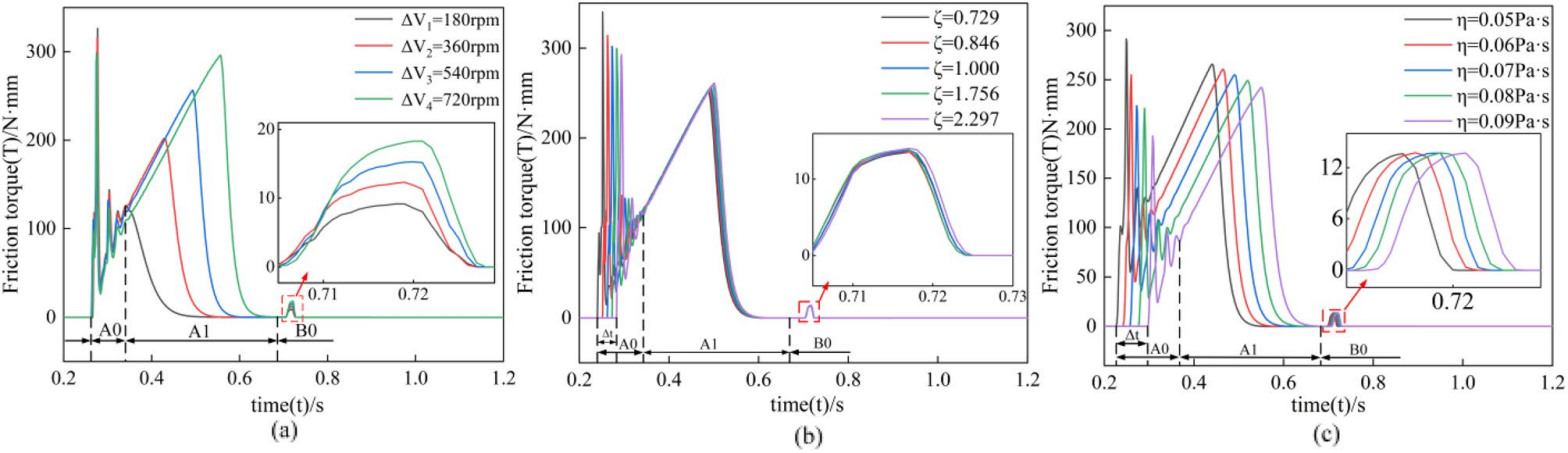
Response of rotational speed difference, damping ratio, and lubricant viscosity to friction torque.

Since clutch engagement and separation are complete processes, clutch engagement is performed first in the simulation setup, and clutch separation is performed when clutch engagement is complete. The friction torque generated by clutch engagement and separation will be analyzed comparatively in the following. In Fig. 7, A0 and A1 represent the touching and slipping processes during engagement, respectively; B0 represents the clutch separation process.

The response of rotational speed difference to friction torque is shown in Fig. 7a. The increase in rotational speed difference decreases the coefficient of dynamics friction and prolongs the time of A1, where the friction torque positively correlates with the rotational speed difference. In A0, the speed difference has no significant effect on the touching time, but decreasing the coefficient of dynamics friction results in a slight decrease in the friction torque at the same time point. During the B0 separation process, the friction pair torque gradually increases with the speed difference; the friction pair contact stress decreases due to the gradual decrease in pressure during separation, and the separation gap subsequently increases. Therefore, in the separation process, the change of rotational speed difference does not affect the friction torque as significantly as in the engagement process.

The response of the damping ratio to friction torque is shown in Fig. 7b. In A0, the increase in the damping ratio reduces the instantaneous speed of the friction pair motion, which decreases the friction torque amplitude. While in A1, the damping change has no significant effect on both friction time and friction speed, although the friction torque is positively correlated with the damping ratio, the growth rate is only 5%, which prolongs the engagement onset time by 7.4% (Δt = 0.043 s). During the B0 separation process, as the damping ratio increases, the motion damping increases, making the lubricant enter the gap for a longer time. Therefore, the friction time is prolonged with the increase of the damping ratio; however, the effect of the change of damping ratio on the friction torque value is insignificant in agreement with the engagement process.

The response of lubricant viscosity to friction torque is shown in Fig. 7c. In A0, as the lubricant viscosity increases, the oil film carrying capacity increases, decreasing the peak friction torque. The increase in lubricant viscosity increases the resistance to motion of the friction pair, reducing the frequency and amplitude of the onset oscillation of the friction torque and delaying the engagement process. Therefore, lubricant viscosity mainly affects the peak value, onset frequency, and fluctuation amplitude of the friction torque and less affect the overall trend. Unlike the joining process in the B0 separation, firstly, the increase in lubricant viscosity reduces the fluidity of the lubricant and prolongs the time of lubricant entry into the gap; secondly, the increase in lubricant viscosity makes the friction pair separation resistance increase, so the change in lubricant viscosity only delays the friction time. Therefore, in the separation process, the response of the lubricant viscosity to the friction torque is consistent with the joining process response, which is small.

### Speed response of friction pair

**Figure 8:**
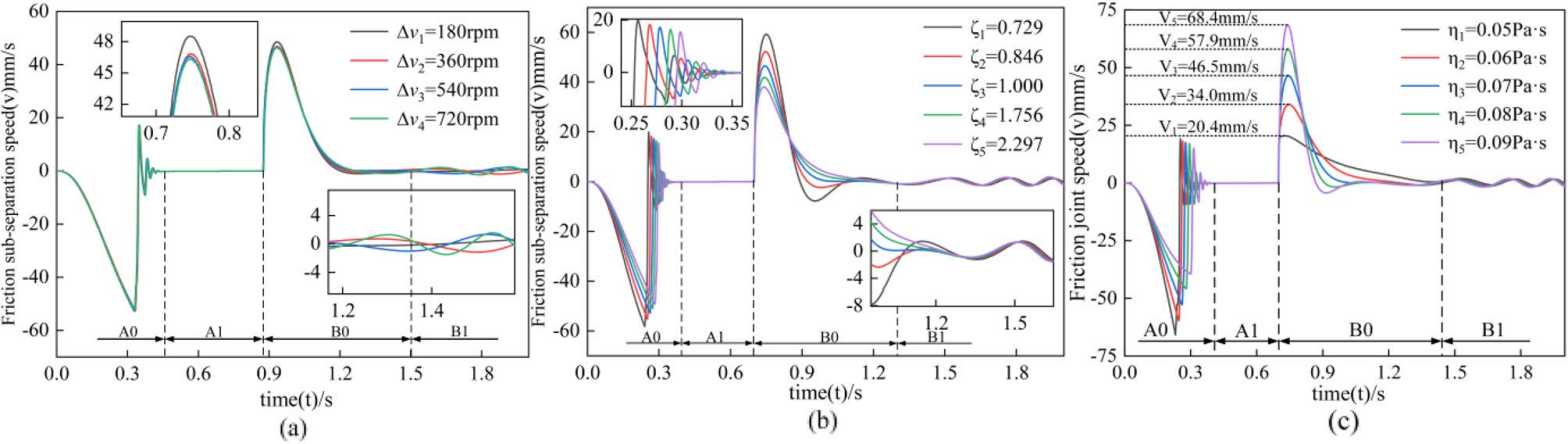
Response of speed difference, damping ratio, and lubricant viscosity to engagement and separation speeds.

The operating speed of the clutch can be divided into two parts, the engagement speed, and the separation speed, which are inextricably linked. This section will compare and analyze the engagement speed and separation speed. In Fig. 8, A0 and B0 represent the engagement and separation processes, respectively, while A1 and B1 represent the engagement completion and separation oscillation processes.

The response of the speed difference to the clutch separation speed is shown in Fig. 8a. With the increase of rotational speed difference, there is no effect on the separation speed in the engagement process A0. In the separation process B0, because the increase of rotational speed difference reduces the dynamic friction coefficient, increases the rotational inertia of the friction plate, and the friction pair momentum increases, the frequency and amplitude of oscillations in the B1 stage increase accordingly. Overall, the change in rotational speed difference has no significant effect on the separation speed.

The damping ratio's response to the clutch's operating speed is shown in Fig. 8b. As the damping ratio increases, the resistance to motion of the friction pair increases, so both the engagement and separation speed decrease. In the engagement process A0, the maximum speed decreases by ten mm/s, and the engagement time is prolonged by 0.044 s. In the separation process B0, the maximum separation speed decreases by 21.1 mm/s, and the magnitude and frequency of the separation speed oscillations decrease due to the decrease in slip acceleration. Therefore, in the underdamped condition (ζ < 1), both the maximum engagement and separation speed of the clutch increased, and in the overdamped condition (ζ > 1), both the maximum engagement and separation speed decreased. However, the damping change had a more significant effect on the separation process B0.

The lubricant viscosity response to the clutch's operating speed is investigated, as shown in Fig. 8c. In the engagement process A0, the increase in lubricant viscosity increases the oil film carrying capacity and resistance to movement, reducing the engagement speed. In the separation process B0, lubricant viscosity increases the Oil film carrying capacity, increasing the separation speed and shortening the separation time. Therefore, the effect of lubricant viscosity on joining speed and separation speed is more significant and plays an opposite role in the joining and separation process.

### Separation gap coefficient of recovery response

In engineering analysis, it is often tricky to deal with the influence of nonlinear factors; the face of the problem is generally used to weaken or ignore the influence of factors; for this problem, this paper establishes the clutch nonlinear dynamic separation model. Since neither the friction torque nor the separation speed can accurately measure the separation end time and the motion state of the friction pair after separation, a gap recovery coefficient was proposed to solve this problem.

**Figure 9:**
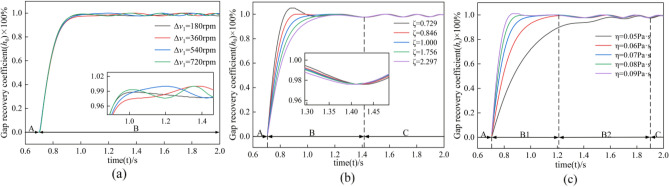
Response of different factors on gap recovery coefficient.

The response of the rotational speed difference to the gap recovery coefficient is shown in Fig. 9a. A indicates the joining process, and B indicates the separation process. The gap recovery coefficient shows a steadily increasing trend because of the insufficient oil film carrying capacity at the initial separation stage. After the gap gradually increases, the oil film carrying capacity increases, and the friction pair changes from unidirectional slip motion to bidirectional fluctuation under the action of inertia and Oil film carrying capacity, so the gap recovery coefficient exhibits an oscillating tendency and finally tends to flatten out under the action of damping. Overall, the change in rotational speed difference only increases the frequency and amplitude difference of the oscillation by a small amount. However, it has no significant effect on the overall change of the gap recovery coefficient.

The effect of the damping ratio on the gap recovery coefficient is shown in Fig. 9b. When the damping ratio ζ > 1, the damping increases the friction pair resistance during the separation process, reducing the separation speed and displacement of the friction pair, so the response of the gap recovery coefficient is slower and reduces the frequency of oscillation during the separation process. When the damping ratio ζ < 1, the motion resistance decreases, the separation speed of the friction pair increases, and the momentum of the friction pair increases, so the gap recovery coefficient responds faster, and the gap recovery time is shortened. Therefore, the damping ratio significantly affects the gap recovery coefficient.

The effect of lubricant viscosity on the gap recovery coefficient is shown in Fig. 9c. With the increase of lubricant viscosity, the Oil film carrying capacity gradually increases, so the gap recovery speed increases, the gap recovery time decreases by 0.086 s, and the separation gap oscillation amplitude tends to level off. Therefore, increasing the lubricant viscosity can shorten the separation time in the clutch separation process, significantly affecting the gap recovery coefficient.

## Friction pair thermal coupling analysis

ABAUQS is utilized to simulate the thermal coupling of the friction pair. Preparation before simulation: Establish the clutch friction pair model, assign the material parameters of the friction plate and steel plate, as shown in Table 2; secondly, divide the hexahedral mesh of the friction pair and set the contact parameters of the friction pair, as shown in Table 2; the piston load is 1.0 MPa, the simulation time is set to 0.3 s, and the step size of the simulation is 3819. To study the effect of different rotational speed differences on the friction pair, two groups of friction pairs are selected for testing. The rotational speed difference is set to 180 rpm, 360 rpm, 540 rpm, and 720  rpm, respectively, to study the effect of rotational speed difference on the friction pair's temperature, contact stress, and pressure field response characteristics. The simulation parameters are derived from the table of working parameters of powershift transmission and related papers, as shown in Table 2 below^31^.

**Table 2:** Parameters of thermally coupled materials.

Steel plate	Initial value	Friction plate	Initial value	Exposure	Initial value
Densities	7850	Densities	2500	Thermal conduction	5.0E+07
Resilient	2.1E + 11	Reachability	0.9		5.0E + 07
Reachability	50	Thermal expansion	1.0E−05 (T = 0)	Coefficient of friction	0.38 (T = 20 °C)
Thermal expansion	1.1E−05		4.0E−05 (T = 200)		0.39 (T = 100 °C)
Specific heat	450	Resilient	2.1E+10 (T = 0 °C)		0.42 (T = 150 °C)
			1.2E+10 (T = 100 °C)		0.39 (T = 200 °C)
			5.0E+09 (T = 200 °C)		0.25 (T > 200 °C)

Marginal notes: friction plate elastic Poisson's ratio (*ν* = 0.25), Poisson's ratio of steel plate elasticity(*ν* = 0.29)^32^.

In the simulation process, to observe the trend and distribution of the temperature and contact stress of the steel plate, the steel plate at Time = 0.08 s, Time = 0.14 s, and Time = 0.2 s are compared and analyzed, respectively. The working environment temperature of the friction pair is set to 20.0 °C in the simulation, and the temperature change of the steel plate is observed under different rotational speed differences, respectively, which are analyzed as follows.

### Temperature field analysis of friction pair

**Figure 10:**
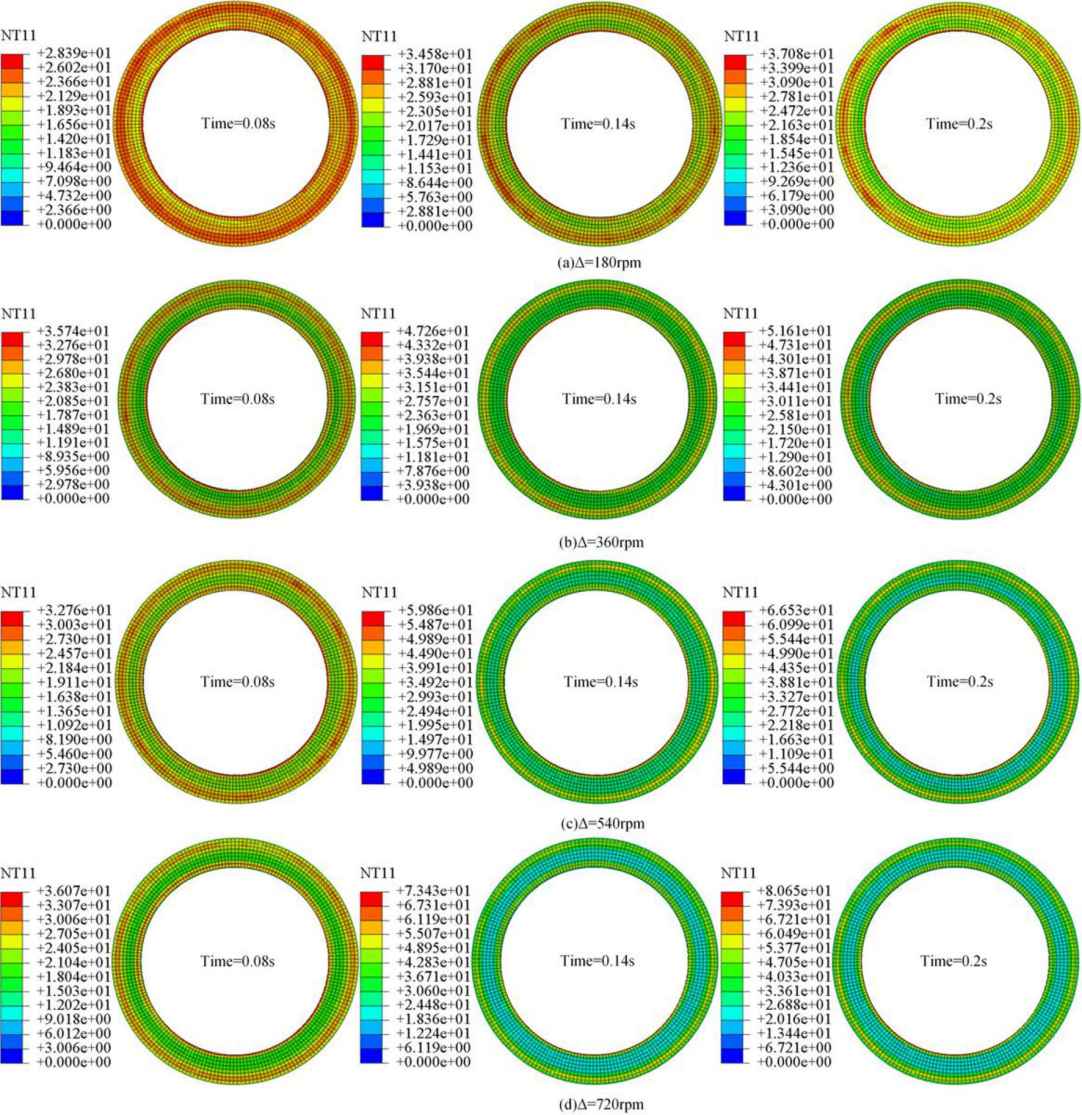
Temperature variation of steel plate at different rotational speed differences.

Figure 10a–d respectively, the steel plate in different speed difference temperature change rule with time. After the friction pair produces the rotational speed difference, because the friction plate outer edge of the line speed is the largest, the outer edge of the steel plate forms a high-temperature annular band; the steel plate inner edge produces high temperature annular is due to the friction plate inner diameter is small with the steel plate internal diameter caused by the friction plate. time = 0.08 s because the friction pair produces a slight rotational speed difference, so the temperature of the steel plate with the rotational speed difference increases only by a slight temperature difference.

As the rotational speed difference continues to increase, the temperature growth rate increases with the increase in rotational speed difference. Due to the decrease in contact stress between the friction pair, the friction pair is only contacted by the micro-convex body, so the contact area of the steel plate is reduced, and the width of the high-temperature annular band decreases with the increase of the rotational speed difference, as shown in Fig. 10 Time = 0.14 s. The temperature difference between the contact surfaces of the steel plate increases with the increase of the rotational speed difference, and the low-temperature band in the middle of the steel plate gradually becomes more comprehensive with the increase of the rotational speed difference, as shown in Fig. 10 Time = 0.2 s.

### Friction pair pressure field

**Figure 11:**
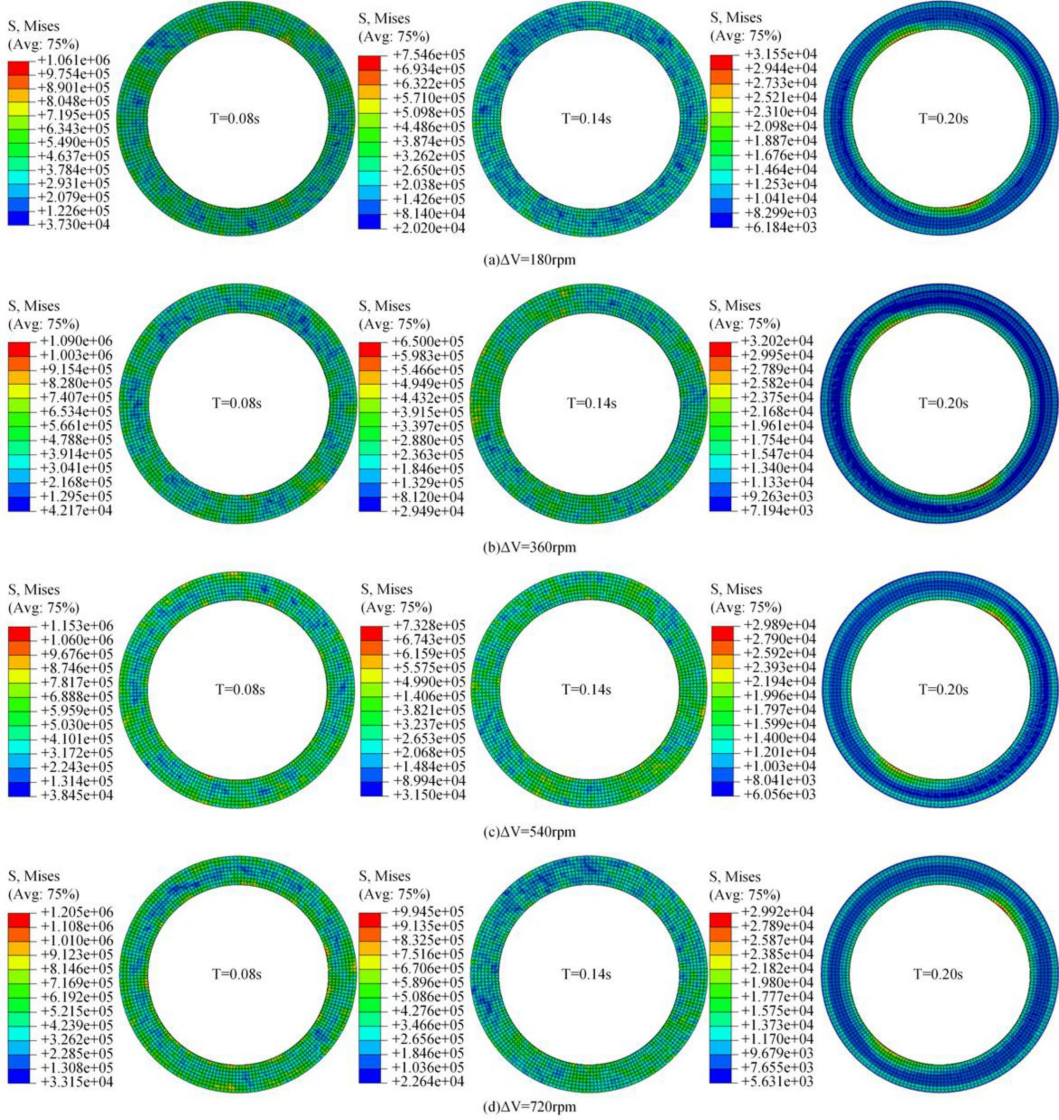
Variation of contact stress of steel plate under different rotational speed differences.

The friction pair steel plate and friction plate were alternately arranged, and two groups of friction pairs were selected to analyze their stress changes. The speed difference is 180–720 rpm, and the contact stress distribution of the friction pair is shown in Fig. 11.

Figure 11a–d shows the variation of contact stress of steel plates at different speed differences, respectively. As shown in Fig. 11a, with the extension of separation time, the contact stress of the steel plate decreases gradually. As the contact stress continues to fall, the stress concentration points on the surface of the steel plate gradually decrease, and the low-pressure point gradually increases. When the contact stress drops to a specific value, a low-pressure annular band is formed in the middle of the steel plate. In the separation process, due to the inconsistency of the inner and outer diameters of the steel plate and the friction plate, the contact stress of the steel plate is mainly concentrated on the outer and inner edges under the influence of the line speed.

## Test analysis

This chapter first introduces the test apparatus and test method, then analyzes the test's torque, temperature, contact stress, and rotational speed, and finally verifies the validity of the simulation model and the correctness of the test results by comparing them with the simulation.

### Test apparatus

**Figure 12:**
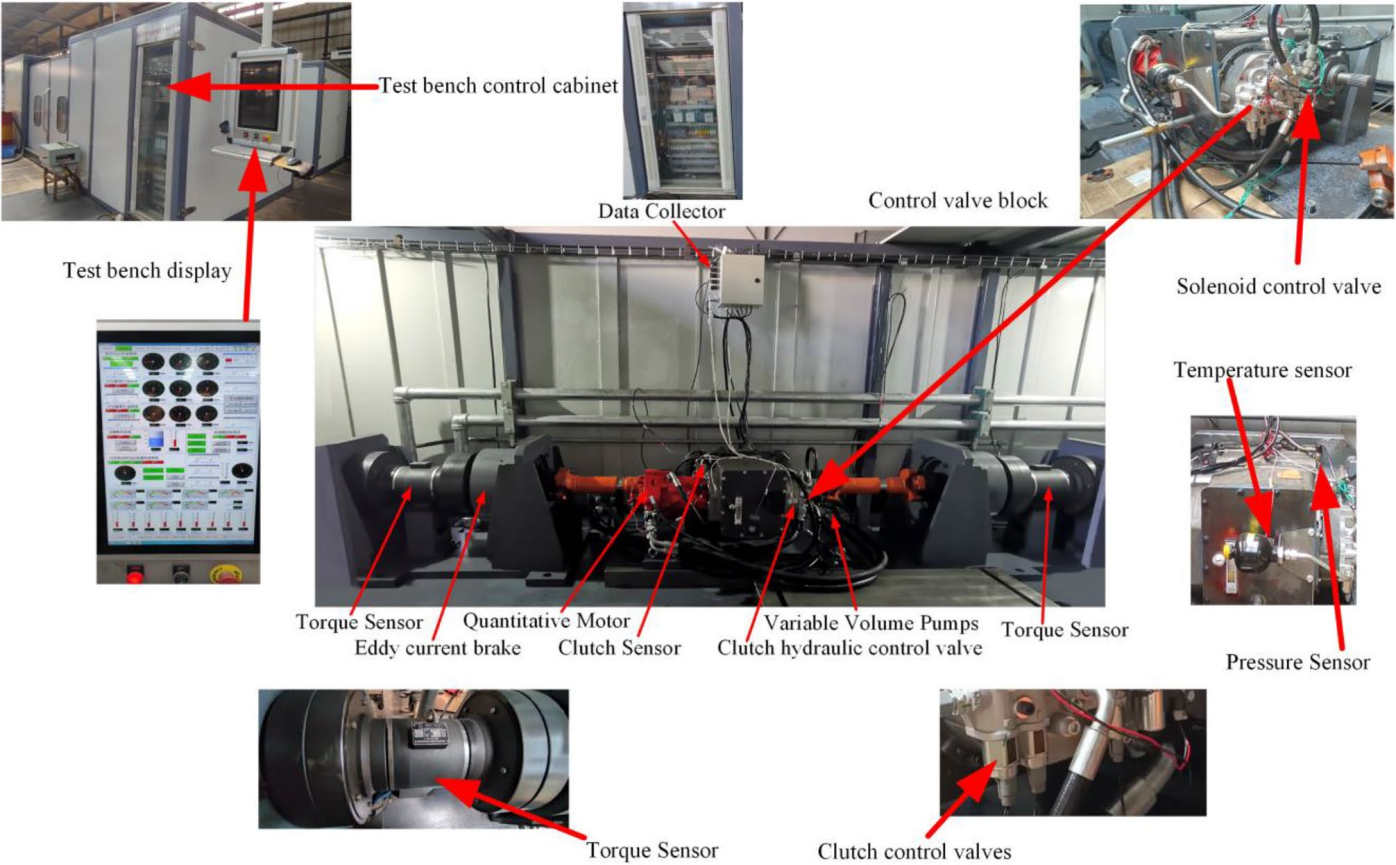
Test bench.

**Figure 13:**
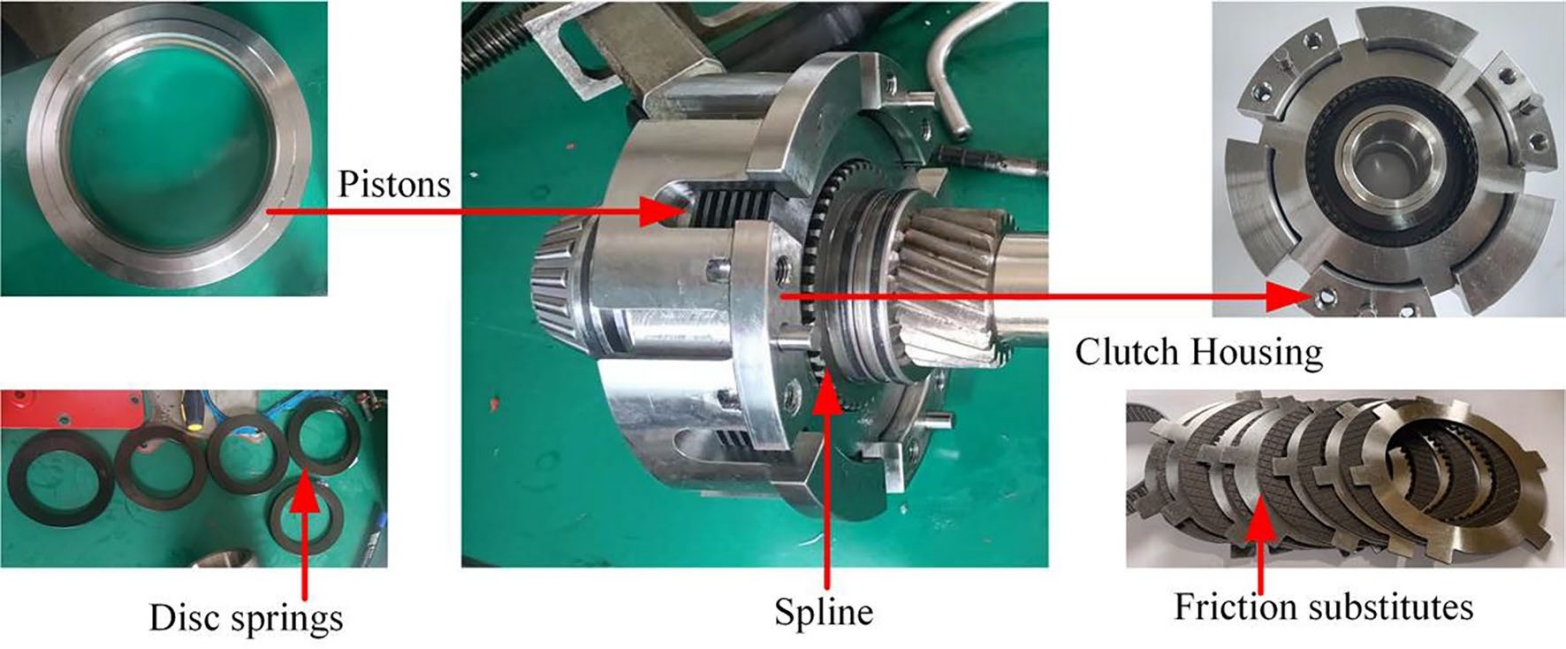
Experimental clutch element.

As shown in Fig. 12, the test bench mainly consists of three parts: transmission part (variable motor, flexible coupling, universal joining coupling, auxiliary pump too, clutch and brake assembly, and electromagnetic reversing valve assembly), measurement and control system (speed and torque sensor, infrared temperature sensor, control cabinet of the test bench, data collector, clutch control valve), and loading equipment (magnetic powder brake). The structure of the test clutch is shown in Fig. 13.

Variable pump as a power source, through the control of the clutch solenoid valve switch, to realize the piston filling and pressure relief, and control the oil pressure size mainly rely on the adjustment of the relief valve to achieve. The variable motor is the power source of mechanical transmission (power of 45 kw, speed range of 0–5000 rpm, torque range of 0–2000 Nm). The test process mainly relies on the frequency converter to adjust its speed and torque. In the test selected, six pieces of friction plate and seven pieces of steel alternating arrangement (friction plate using 30CrMnSi paper-based friction material, friction plate OD 130.9 mm ID 101 mm steel plate OD 132.8 mm steel plate ID 98.4 mm steel plate thickness 1.6 mm). The primary sensors.

are infrared temperature sensor D4060A (measuring range − 60 °C to − 380 °C, response time 300 ms), pressure sensor FS (sensitivity 0.36% sampling frequency 9600 Hz precision 0.05 N), speed and torque sensor TR-1C (torque collection range 0–1000 Nm, speed collection range 1.2–6000 r/min, speed and torque collection error are both 0.2–1000 Nm. and torque acquisition error are 0.1%), magnetic particle brake CZ-500 (slip power of 45 kw, rated torque of 5000 Nm). The test data were measured by the above sensors and imported into the computer by the collector.

### Test methods

At the beginning of the experiment, the clutch housing is first filled with oil (type MOTUL) to ensure the cooling and lubrication conditions of the wet clutch. Then, connect the universal joining coupling with the output shaft and install the variable pump and motor to form a complete transmission system. When the test bench is installed, the first 30-min pre-test will be completed after the test bench pre-test and then the clutch release test.

**Figure 14:**
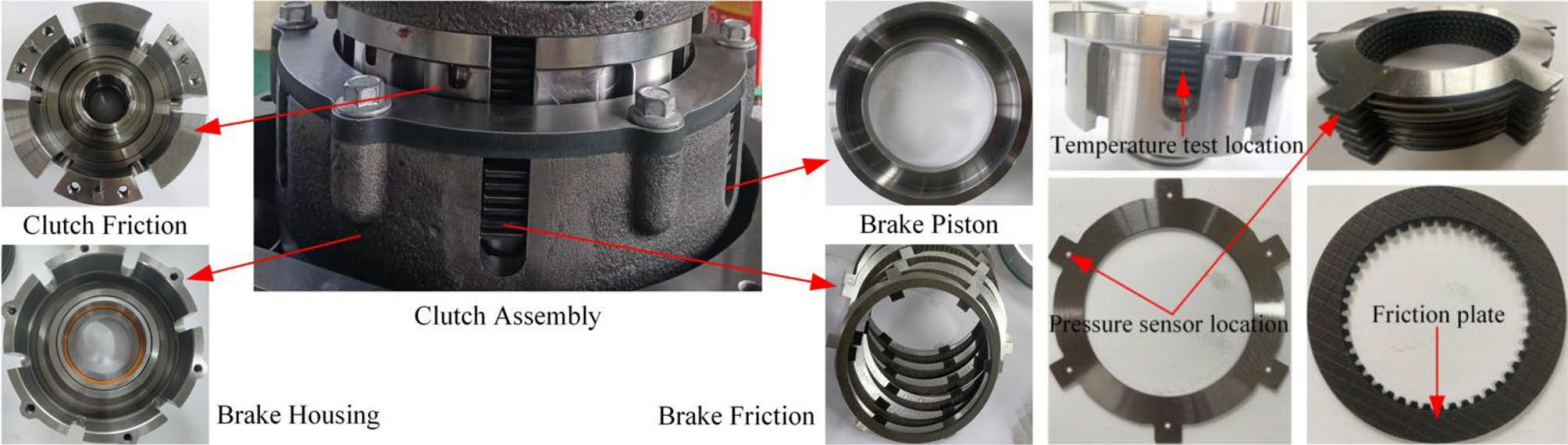
Clutch test parts.

**Figure 15:**
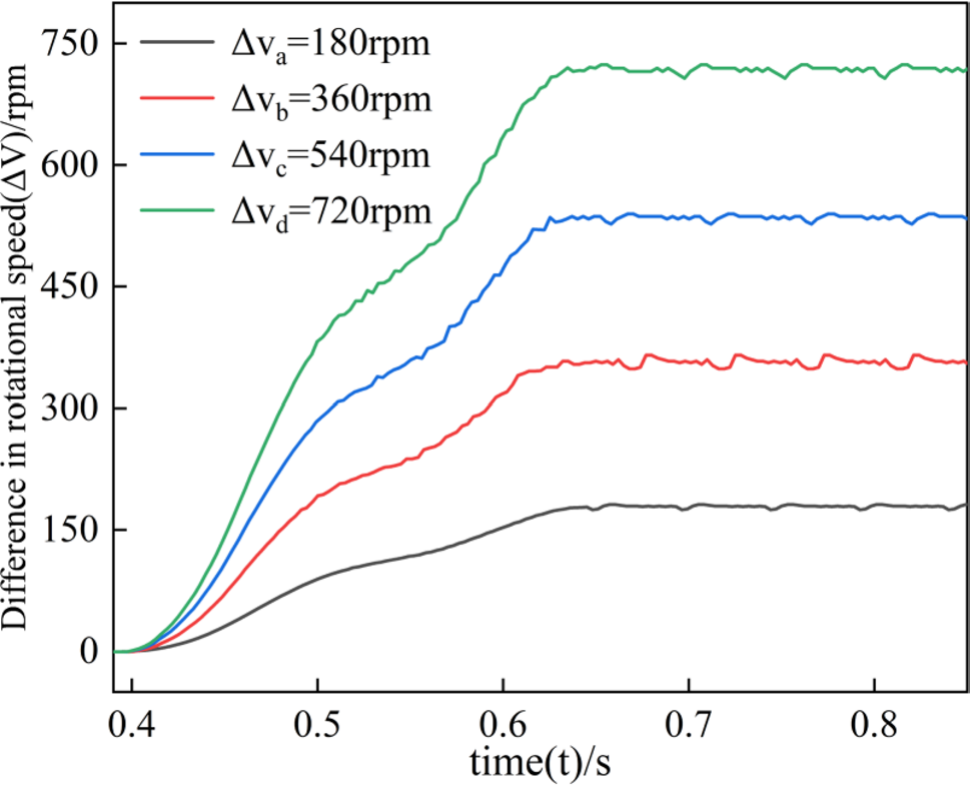
Experimental equipment speed difference loading test.

First, the brake is fixed by a solenoid valve, as shown in Fig. 14. The rotational speed difference is shown in Fig. 15, and the change of rotational speed and torque under different rotational speed differences is tested, in which each group of tests is measured four times respectively. In the temperature test, because the friction plate is a rotating active part, it is impossible to measure the temperature of the same position continuously, so we choose the steel tabs as the measurement object; the tabs are uniformly distributed at 60° in 6 different areas, due to the restriction of the sensor position, so we can only choose one tab as the test object, the four groups of experimental data in the order of a, b, c, and d.

In the pressure test, six different areas spaced at 60° intervals on the contact surface of the steel plate are selected as test points. The test data from each of the two different locations in each group of tests are sorted as a-h. Finally, the test data are outputted to the test bench display using sensors, data acquisition, and other measurement and control elements.

### Experimental analysis

**Figure 16:**
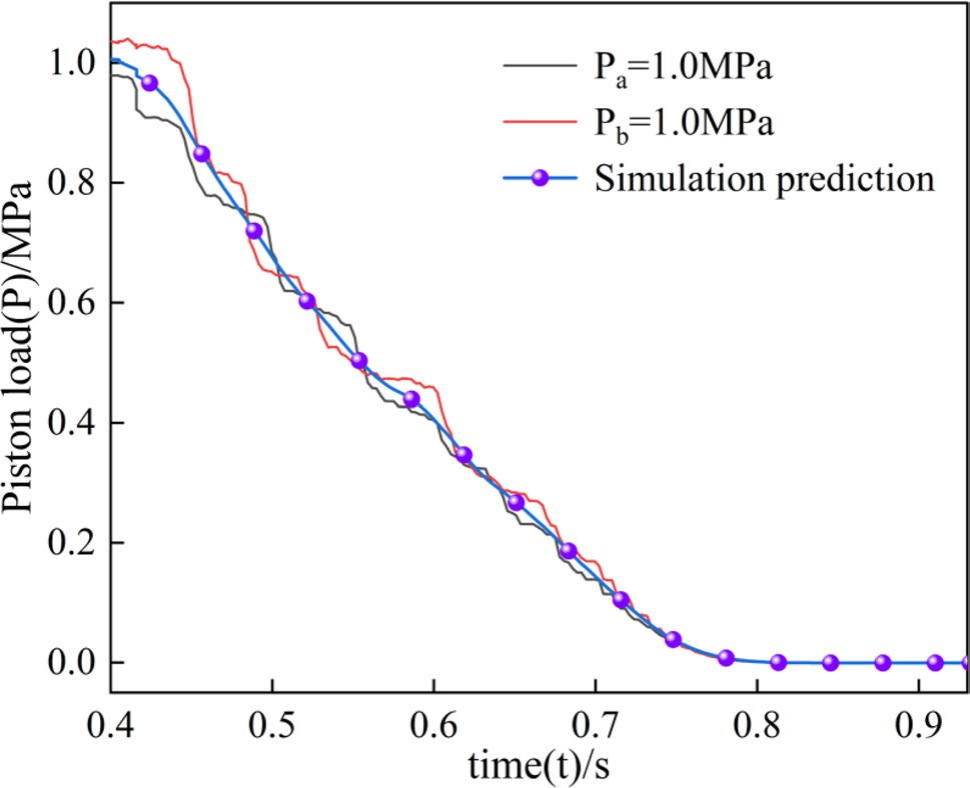
Piston load variation.

The piston load in both test and simulation is 1.0 MPa, and in the separation process, the clutch solenoid valve controls the clutch pressure charging and pressure relief. In the pressure relief process, the oil pressure change is not transient but a gradual and slow decline, as shown in Fig. 16. Since the oil pressure is dynamically changing in the test and fluctuating around 1.0 MPa, the simulation and the test of the piston load in the trend of the change in the same but in the value of the slight differences.

**Figure 17:**
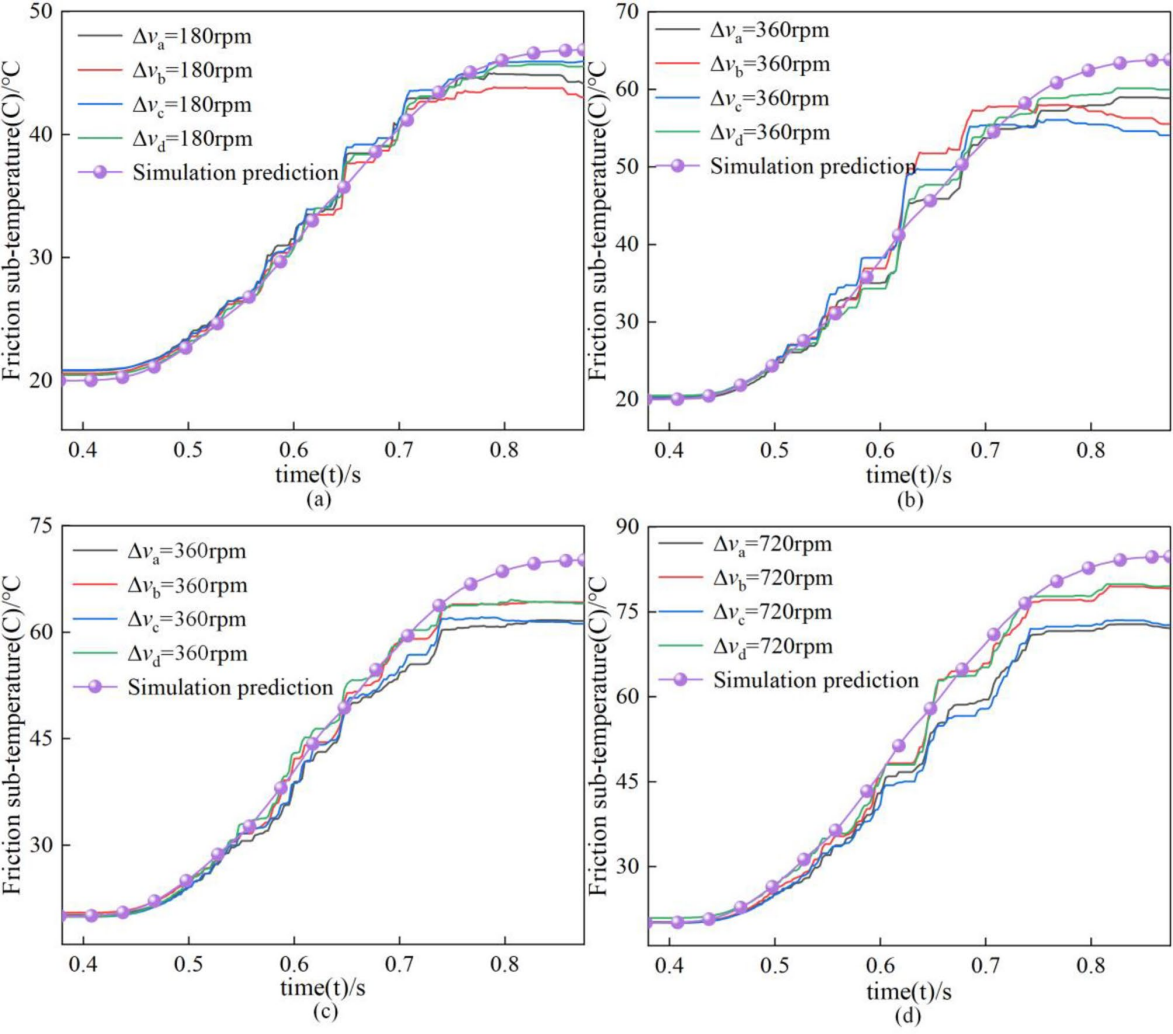
Temperature test of friction pair steel plate at different speed differences.

Figure 17 shows the temperature variation curves of the steel plate at different speed differences. The temperature of the steel plate shows a slow increase, then stabilizes and finally decreases. It is worth noting that the temperatures of the steel plate in the test are lower than the simulation temperature values. In addition, the temperature change trend of different areas of the steel plate is consistent, but the temperature values are different, and the maximum temperature difference is ΔC = 9.7 °C. By comparing the test and simulation results, it was found that the more significant the speed difference, the more significant the temperature difference between the test and simulation temperature prediction curves. This is first because in the simulation, the influence of the spline and the clutch housing on the temperature is not considered, and in the test, both the spline and the clutch housing can transfer heat. Secondly, the simulation does not consider the heat carried away by the lubricating oil. Finally, in the test, due to the nested arrangement of the two clutches working at the same time, as shown in Fig. 14, the mutual influence of the temperature in the process of engagement and separation leads to the inaccuracy of the temperature in the experimental test, so there are differences in the temperature curves of different groups of experiments. Therefore, the experimental results are closer to the simulation results when the rotational speed is lower; when the rotational speed difference is more significant, the simulation only predicts the range of temperature change.

**Figure 18:**
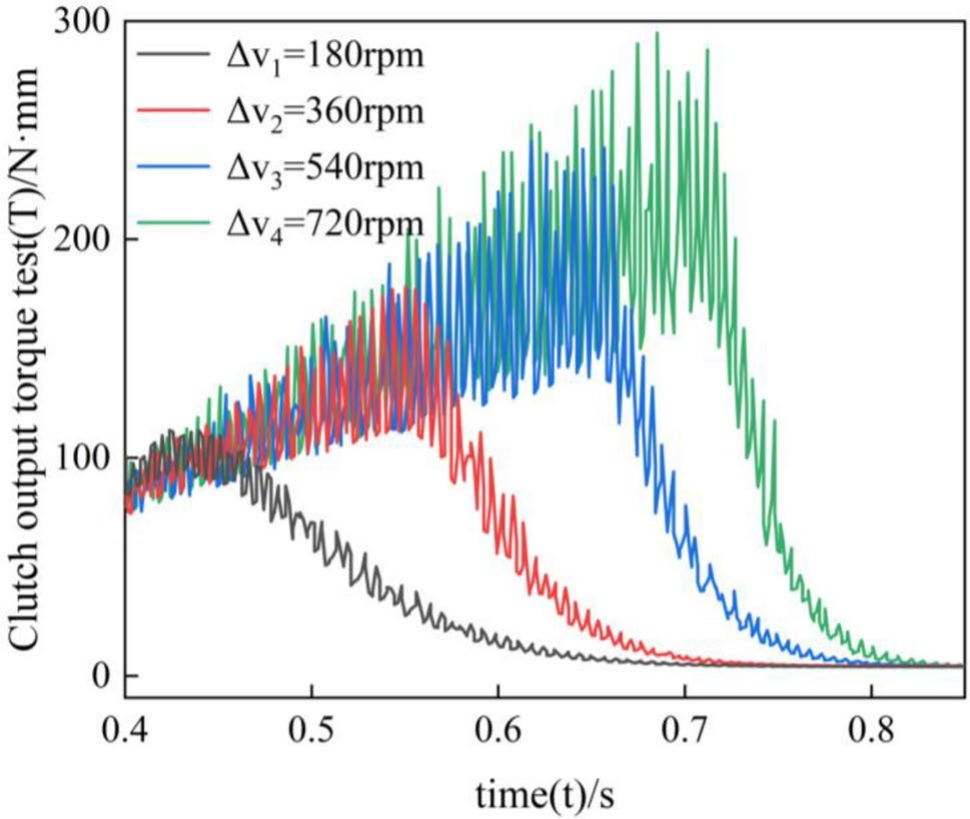
Clutch torque output at different speed differences.

As shown in Fig. 18, it is the clutch separation torque test. During the separation process, the speed difference increases with time. However, the friction force decreases as the separation gap increases, and when the separation gap returns to a specific value, there is no friction between the friction pair. Hence, the friction torque shows a tendency to increase and then decrease. In the separation process, the friction plate and the steel plate are not just unidirectional movements; the contact stress is not a continuous value, so the instantaneous torque always presents an oscillating state. Similarly, due to the instability of the slip acceleration and the sudden change of the contact stress, the frequency and amplitude of the torque oscillation also increase with the increase of the rotational speed difference. By comparing the experimental and simulation results, it is found that the two trends are consistent with the first increase and then decrease, but there are large differences in the values.

The measurement error dramatically impacts the results because the friction time in the separation process is very short. Secondly, in the test, two groups of clutches work in the opposite process; in the test of a group of clutch separation at the same time, the other group in the engagement, as shown in Fig. 14; the existing experimental conditions cannot be individually measured in a group of clutch friction torque, so the test results in the numerical comparison with the simulation has a significant deviation.

**Figure 19:**
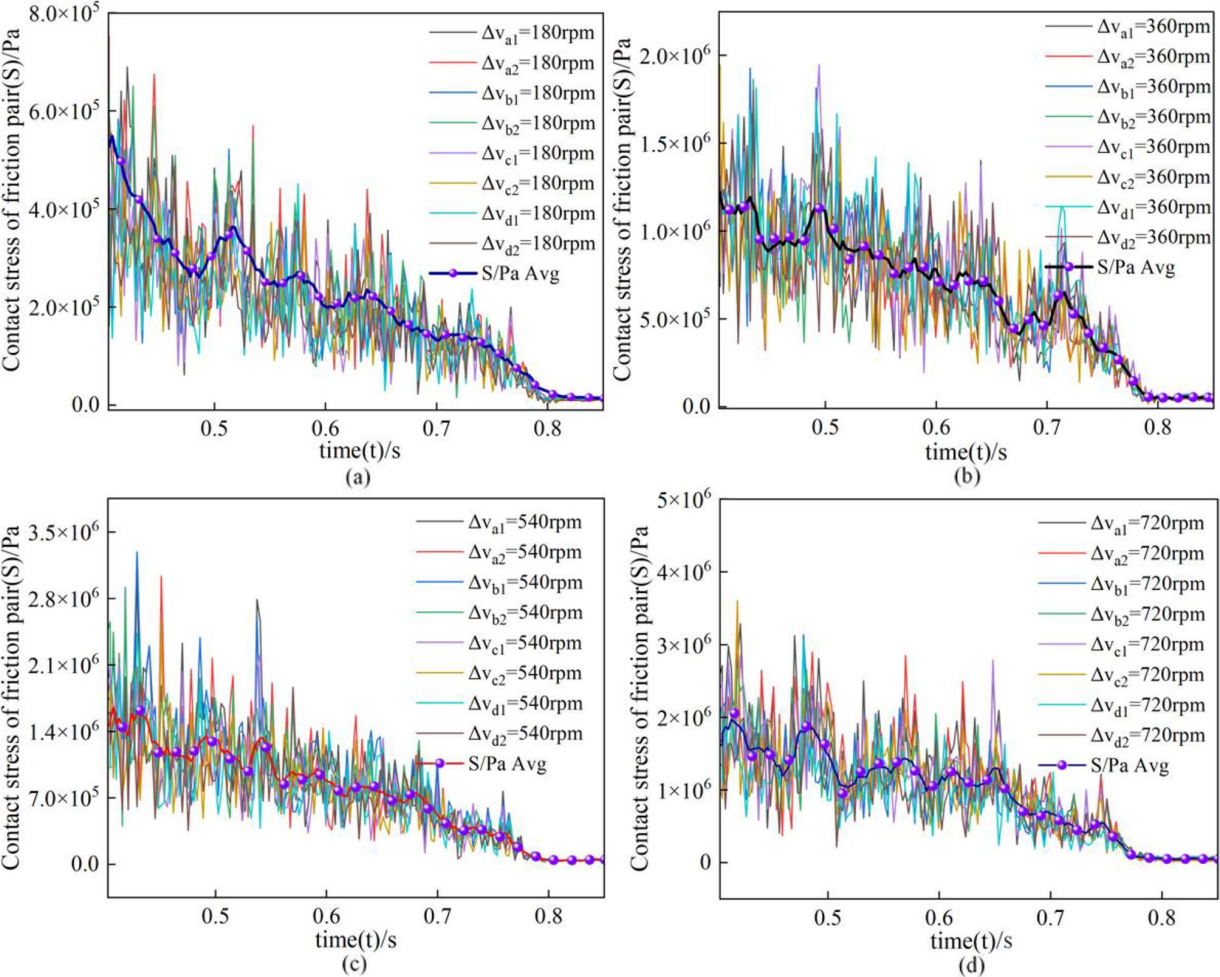
Contact stress of steel plate at different rotational speed differences.

As shown in Fig. 19, the stress change of steel plate under different rotational speed differences. In the legend, a, b, c, and d denote the measurement results at different positions; the numbers 1,2 denote the first test and the second test, respectively, and the S/Pa Avg curve denotes the mean line; ΔV denotes the rotational speed difference of the friction pair; and Fig. 19a–d denotes the change of contact stress of the steel plate when the rotational speed difference is increased from 180 to 720 rpm, respectively.

By comparing the changes of contact stress under different speed differences, it is found that the mean value of stress decreases with the extension of separation time due to the uneven contact between the friction pair and the uncertainty of the friction plate movement in the separation process, which leads to different contact stress in different areas of the steel plate with a more significant frequency of oscillation. However, it can be found from the mean value of stress that the trend of contact stress is consistent with the simulation; with the increase of rotational speed difference, the stress values all show a positive correlation from 5.6 × 10^5^ to 1.8 × 10^6^ Pa.

A comparison of simulation and test results shows differences in the results between simulation and test due to the variability of test conditions and the insufficiency of simulation. However, the test and simulation results are consistent regarding the changing trend. They can predict the form of change of the separation process under certain conditions, which also verifies the validity of the simulation model.

## Conclusion

This paper aims to reduce the friction pair erosion phenomenon of the friction pair of the motorized clutch in the case of continuous shifting; it establishes a theoretical model of the motion process of the friction pair and deduces the relationship between the friction torque, the speed difference, the friction time, and the heat generation through the friction pair heat generation model. Therefore, the rotational speed difference, friction torque, and separation speed were selected as the research objects of separation characteristics, and a nonlinear dynamics simulation model was established for them to study the effects of different factors on friction torque and separation speed during the separation process. During the separation process, since neither the friction torque nor the separation speed can accurately measure the separation end time and the motion state of the friction pair after separation, a gap recovery coefficient is proposed to solve this problem. Given that the dynamics simulation model has defects in temperature and flexible body, a finite element thermodynamic coupling model is established to fill this deficiency. Finally, the clutch separation test verified the simulation model's validity. The following conclusions can be drawn from the appeal study:

1. The rotational speed difference has the most significant influence on the temperature of the friction pair, and lowering the rotational speed difference can effectively reduce the heat generation of the friction pair, in which the rotational speed difference mainly affects the temperature by changing the friction torque. However, the uniformity of the contact stress distribution of the friction pair in the separation process is unrelated to the speed difference. Therefore, in the actual working conditions, we should choose the low-speed interval for gear shifting to avoid friction pair erosion caused by frequent gear shifting in the high-speed interval.
2. The lubricant viscosity has the most significant effect on the separation time and speed. Increasing the lubricant viscosity by 0.04 Pa s can shorten the separation time by 27.71% and increase the maximum separation speed by 21.9 mm/s. Therefore, increasing the lubricant viscosity can reduce the heat generated by the friction pair.
3. Damping is positively correlated with the heat generated by the friction pair. Reducing the damping ratio by 1.568 can shorten the separation time by 11.42% and increase the maximum separation speed of the friction pair by 22.3 mm/s. Therefore, the damping of the friction pair should be minimized to reduce the heat generated by the friction pair in practical work.

To summarize, for the problem of clutch friction pair erosion in the continuous shift process, the lubricant viscosity in the clutch should be increased, and the friction pair rotational speed difference and motion damping can be reduced to minimize the heat generation of the friction pair and avoid the friction pair erosion phenomenon. In future research, we should also focus on constructing a lubricant hydrodynamic model and lubricant heat transfer model to study the friction pair erosion phenomenon further.

## Data Availability

The datasets used or analyzed during the current study are available from the corresponding author on reasonable request.
